# Supplementary material for: Intra-Host Evolution of SARS-CoV-2 During Persistent Infection of Pediatric COVID-19 Patients
Source: Viruses. 2025 Sep 28;17(10):1313. doi: 10.3390/v17101313 (PMC12567731; doi:10.3390/v17101313)
Supplement: Supplementary file 1 [file viruses-17-01313-s001.zip › viruses-3861865-Supplementary Materials.pdf]

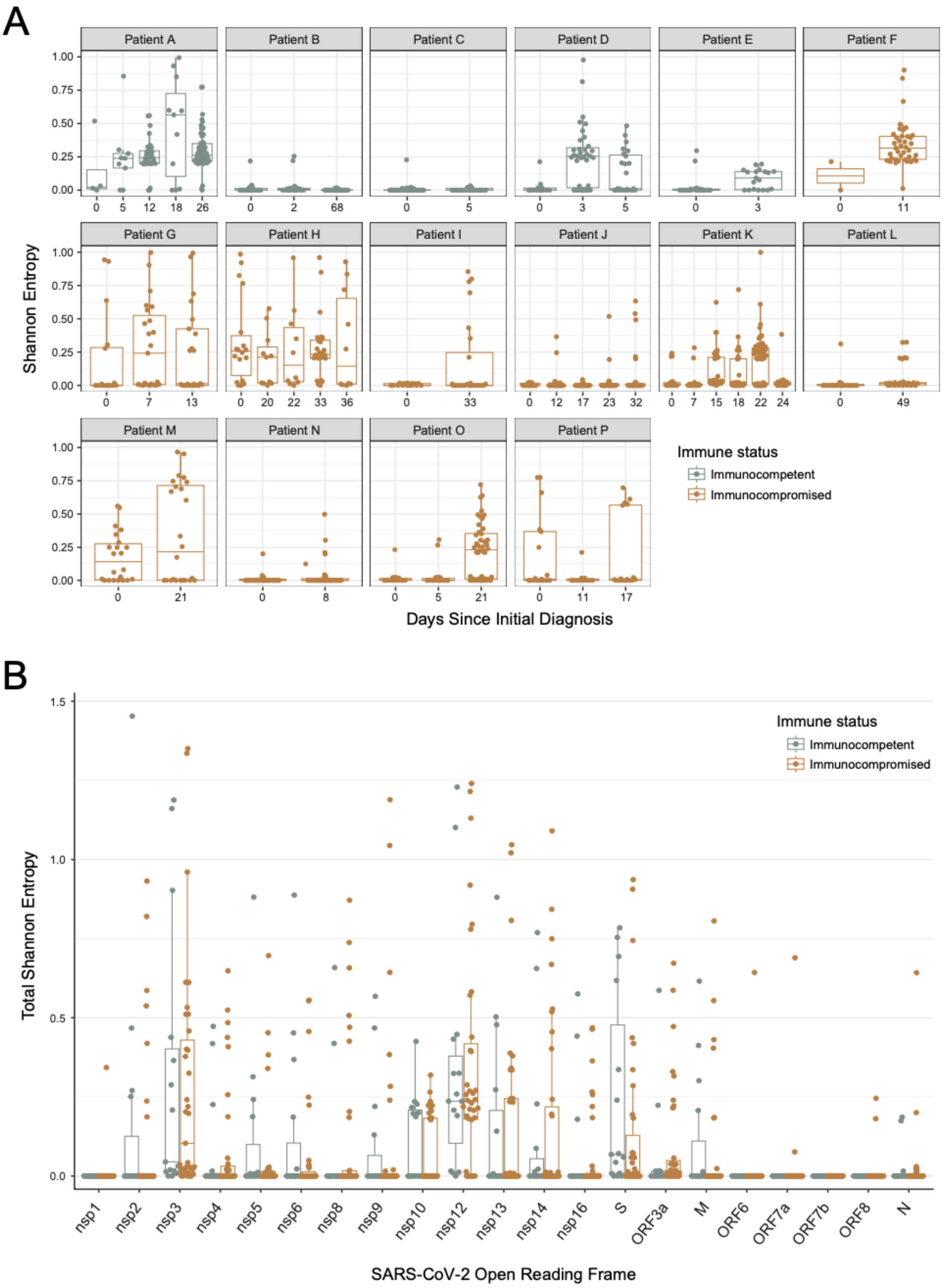

Supplemental Figure S1 | Intra-host diversity of SARS-CoV-2 by patient and open reading frame. A) Box plots of Shannon entropy values for each position in a given specimen across longitudinal time points for each patient (immunocompetent patients in blue, A-E; immunocompromised patients in orange, F-P). For this analysis, each genomic site with detectable diversity as defined by positions with a read depth greater than or equal to the lower bound across all timepoints within a patient were included (lower bound calculated as 90% of the smallest depth value exceeding 400 reads). **B)** Shannon entropy

per patient sample within each viral open reading frame, comparing immunocompetent and immunocompromised patients. *Sh* values per position were imputed as 0 for positions that did not pass the 3% mutational frequency threshold but have a depth greater than or equal to lower bound to avoid group imbalance in modeling. The lower bound was calculated as 90% of the smallest depth value within the same position across the dataset that exceeds 200 reads. Statistical analysis was conducted using a linear mixed-effects model while controlling for Ct value and patient-level random effects. P-values were adjusted for false discovery rate (FDR) using the Benjamini-Hochberg method. P-values were not significant. For (A) and (B), boxplots depict median and interquartile ranges (IQR), with tails extending 1.5 times the IQR.

SUPPLEMENTAL TABLES

Supplemental Table S1 | GISAID and NCBI Accession numbers for deposited sequences in this study.

Supplemental Table S2 | Mutational frequency at positions in Spike. Only positions that changed more than 3% but less than 97% at any given timepoint in any patient in comparison to SARS-CoV-2 reference sequence (accession MN908947.3) are represented. Mutational frequencies of 0 are not shown. Supplemental table is located in GitHub at https://github.com/Sakazakii/Intra-Host-Evolution-of-SARS-CoV-2-during-Persistent-Infection-of-Pediatric-COVID-19-Patients.
